# Assessment of a new unidirectional barbed suture versus a conventional suture for anastomosis during robotic-assisted Roux-en-Y gastric bypass surgery in obese patients: BARGASTRO — study protocol for a German randomized active-controlled trial

**DOI:** 10.1186/s13063-025-09401-9

**Published:** 2026-01-06

**Authors:** Jan Henrik Beckmann, Petra Baumann, Charlotte Jackisch, Florian Richter, Anne Sophie Mehdorn, Thomas Becker, Terbish Taivankhuu, Witigo von Schönfels

**Affiliations:** 1 Department of General, Visceral, Thoracic, Transplant and Paediatric Surgery, University Hospital Kiel, Arnold-Heller Strasse 3, Kiel, 24105 Germany; 2 Department of Medical Scientific Affairs, Aesculap AG, Tuttlingen, 78532 Germany

**Keywords:** Barbed suture, Bariatric surgery, Anastomosis, Robotic-assisted surgery, Roux-en-Y gastric bypass, Complications, Suturing time, Obesity

## Abstract

**Background:**

Gastric bypass surgery is an efficient surgical intervention for the treatment of obese patients. The procedure can be performed either laparoscopically or robotic assisted. To simplify the intracorporeal handling of the suture material in a bi- or tri-dimensional plane, a new generation of sutures, named barbed sutures, has been developed recently and is being used for different indications. Barbed sutures allow knotless slippage-resistant suturing due to the self-anchoring elements distributed along the thread length and therefore obviate the need for assistance. Their safety and efficacy have been demonstrated in several meta-analyses and systematic reviews. In the present study, the clinical performance of a new unidirectional barbed suture material, which differs in regard to its configuration from other barbed suture materials, is analysed and compared to a conventional suture material in robotic-assisted gastric bypass surgery to treat morbid obesity.

**Methods:**

The study will be performed as an industry-sponsored, mono-centre, randomized, active-controlled, two-arm, parallel-group, superiority trial. A total of 150 patients will be randomly allocated to both suture types in a 1:1 ratio. The suture material will be used to construct the gastro-jejunal anastomosis as well as the jejuno-jejunal anastomosis. The time needed to create both anastomoses is the primary endpoint. Intraoperative handling of both suture materials, patient satisfaction, pain, and complications occurring during the study will serve as secondary outcomes. Furthermore, the difference in quality of life using two different questionnaires (EQ5D5L and Bariatric Analysis and Reporting Outcome System (BAROS)) will be assessed preoperatively and postoperatively (30 days and 12 months) and will be compared between the suture groups as well as over time. The EQ-5D-5L questionnaire consists of five dimensions (mobility, self-care, usual activities, pain or discomfort, anxiety or depression) and a visual analogue scale (VAS) to record the patient’s health status. The BAROS evaluates three sub-categories: The percentage of excess weight loss, the changes in medical conditions, and quality of life, leading to three individual sub-scores, which are added to the final total score defining the failure or success of treatment. The follow-up period is 1 year after surgery.

**Discussion:**

To our knowledge, the BARGASTRO study will be the largest randomized controlled trial investigating a barbed suture versus a conventional one in robotic-assisted gastric bypass surgery to treat obese patients. The study will provide and report clinical data on evidence level Ib.

**Trial registration:**

NCT05433688. Registered on 12 Oct. 2022

## Administrative information

Note: The numbers in curly brackets in this protocol refer to SPIRIT checklist item numbers. The order of the items has been modified to group similar items (see http://www.equator-network.org/reporting-guidelines/spirit-2013-statement-defining-standard-protocol-items-for-clinical-trials/).

**Table:** 

Title {1}	Assessment of a new unidirectional barbed versus conventional sutures for anastomosis during robotic-assisted gastric bypass surgery in obese patients: BARGASTRO – study protocol for a German randomised, active-controlled trial
Trial registration {2a and 2b}.	NCT05433688, www.clinicaltrials.gov
Protocol version {3}	BARGASTRO_CIP_Date 12-03-2021, Version 1.0.
Funding {4}	B. Braun Surgical SA has sponsored and funded the study. In addition, the suture material used during interventions was provided by the sponsor. The sponsor will take over the costs for open-access publication.
Author details {5a}	Jan Henrik Beckmann^1^, Petra Baumann^2^, Charlotte Jackisch^1^, Terbish Taivankhuu^1^ Florian Richter^1^, Anne Sophie Mehdorn^1^, Thomas Becker^1^, Witigo von Schönfels^1^ ^1^University Hospital Kiel, Department of General, Visceral, Thoracic, Transplant and Paediatric Surgery, Arnold-Heller Strasse 3, 24105 Kiel, Germany ^2^Aesculap AG, Department of Medical Scientific Affairs, 78532 Tuttlingen, Germany.
Name and contact information for the trial sponsor {5b}	B. Braun Surgical SA, Carretera de Terrassa 121, 8191 Rubi, Barcelona, Spain.
Role of sponsor {5c}	The sponsor has funded and sponsored the study. The sponsor has made the decision to submit the study protocol for publication. The Department of Medical Scientific Affairs of the company Aesculap AG was responsible for study design, project management, monitoring, data management and statistics and has the ultimate authority over these activities. The sponsor of the study B. Braun Surgical SA is a subsidiary of Aesculap AG.

## Introduction

### Background and rationale {6a}

Laparoscopic gastric surgery (LGS) is the standard surgical treatment for gastric cancer and obese patients [1]. Known short-term complications after LGS are anastomosis leakage, bleeding, or obstruction, which may lead to ileus or peritonitis requiring a re-intervention, causing a longer hospital stay. Therefore, a secure creation of an intestinal anastomosis is of major importance. Different devices, such as staplers or suture materials applied either in the interrupted or continuous suture technique, are currently in use to create an anastomosis [2, 3].

In laparoscopic surgeries, lengthened surgical times are mainly due to three factors: first, the difficulty of working in a bidimensional plane; second, the identification of the surgical planes; and, third, the handling of intracorporeal and extracorporeal sutures. The latter is being the most difficult for surgeons in training when the whole procedure must be achieved by taking the laparoscopic approach [2–5]. Barbed sutures have been reported in the literature to greatly facilitate the suturing during different laparoscopic procedures compared to traditional sutures [6–9].

Barbed sutures are available as absorbable (mid-term and long term) as well as non-absorbable monofilament sutures. The barbs are either arranged in a unidirectional or bidirectional configuration and allow knotless slippage-resistant suturing due to the self-anchoring elements distributed along the suture length, which penetrate the tissue and lock the suture in place [10]. By eliminating knots, there is an inherent reduction in inflammation due to the reduction of foreign material at the wound site [11–13]. This type of suture is nowadays frequently used in gynaecological, urological, orthopaedic, and aesthetic surgical procedures [14–20]. Studies comparing barbed threads versus traditional suture material have shown equivalence regarding performance, safety, and tensile strength [15, 20]. The benefits of barbed sutures are the easy usage and the speed of placement [11, 13]. In addition, this type of suture material provides a uniform tension along the closure line and multilayer closure is possible [21, 22]. Barbed sutures obviate the need for assistance and lead to increased suture efficiency, especially with tissue under tension. The suture material has the potential for less scarring [23], reducing blood loss and surgical time, and knot complications can be avoided [6, 7, 13].

Several studies exist that compare barbed sutures to traditional sutures for the construction of intestinal anastomosis during laparoscopic gastric surgery [6–9]. De Blasi and co-workers observed a reduced anastomosis suturing time and a decrease of operation costs in patients undergoing a laparoscopic Roux-en-Y bypass surgery compared to traditional sutures without an increase in the complication rate [24]. Similar findings were reported by Gys et al., Milone et al., and Constantino et al. [25–27]. The usage of barbed suture for laparoscopic RYGB revealed a faster enterotomy closure, lower costs, a similar complication rate, and no difference in overall operation duration in comparison to conventional sutures. The outcome of the literature showed that barbed sutures are safe and effective for wound closure with a comparable complication rate, which indicates that their performance is at least equivalent to conventional sutures for soft tissue approximation [6–9].

Currently, less clinical evidence is available regarding the role of barbed sutures used for anastomosis formation during robotic-assisted bariatric surgery, notwithstanding their usage is already widespread in this surgical intervention. In the authors’ view, barbed sutures have great advantages especially in robotic bariatric surgery, because in contrast to laparoscopic settings no assistance has to control, pull, and tighten the thread regularly, which simplifies the handling and construction of the anastomosis.

At the time, when the study protocol was prepared for the present study, a total of 14 clinical studies had been performed prospectively or retrospectively, either comparing conventional suture materials to barbed sutures [24–26] or reporting the outcome of barbed sutures [17, 28–36] for the construction of intestinal anastomosis during laparoscopic gastric surgery using either unidirectional or bidirectional mid-term or long-term barbed sutures. To our knowledge, the current RCT “BARGASTRO” will enrol the highest number of patients ever systematically analysed and published in robotic-assisted gastric bypass surgery. Furthermore, it will provide clinical data on evidence level Ib, due to its design. The present industry-initiated study is performed after the CE-mark of the suture material, and the participating hospital is the first user of the new unidirectional barbed suture in general surgery.

### Objectives {7}

The study aims to show the superiority of a new barbed suture over a conventional suture material regarding the suturing time needed to perform the gastro-jejunal (GJA) and jejuno-jejunal anastomosis (JJA) after robotic-assisted Roux-en-Y gastric bypass surgery (RYGB) in bariatric patients, without an increase in the complication rate.

### Trial design {8}

**Fig. 1:**
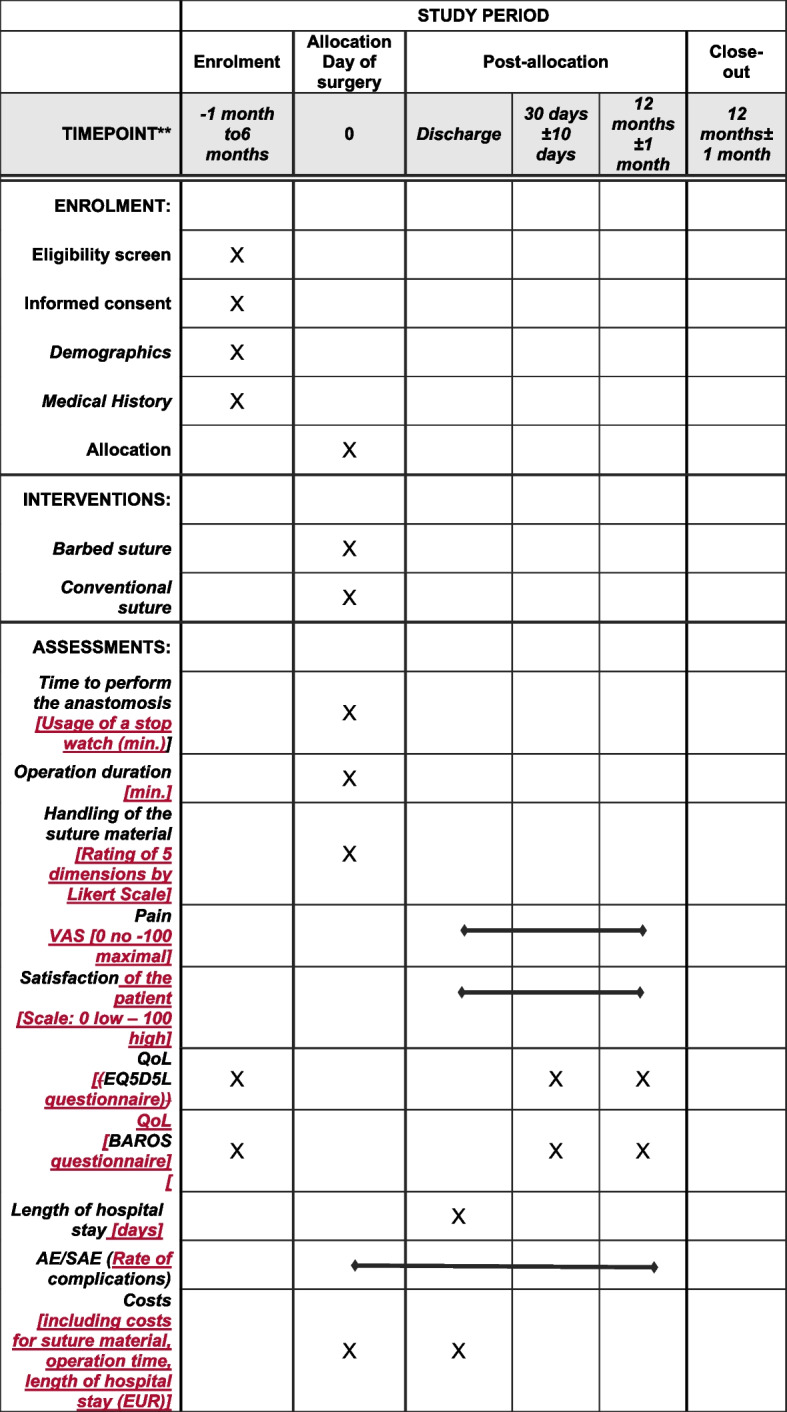
SPIRIT diagram for BARGASTRO. Legend: AE: Adverse Event,: BAROS: Bariatric Analysis and Reporting Outcome System, QoL: Quality of Life, VAS: Visual Analogue Scale, SAE: Serious Adverse Event, Handling assessment of the suture material by the surgeon regarding the different dimensions using a Likert Scale (1 excellent, 2 very good, 3 good, 4 satisfied, 5 poor). In the case of conventional suture the following dimensions will be assessed: pliability, tissue drag, knot security, knot run down, knot pull tensile strength, anastomosis approximation, overall impression. In case of the barbed suture material the dimensions: pliability, pass through the tissue, traumaticity of the barbs, anchoring capacity of the barbs, perception of the locking system, anastomosis approximation, overall impression will be judged. EQ5D5L questionnaire consists of 2 parts - a descriptive system including the following 5 dimensions: mobility, self-care, usual activities, pain or discomfort, anxiety or depression, which are rated on a 5 point level scale (1=no problem, 2=slight problems, 3=moderate problems, 4=severe problems and 5= extreme problems), - and the EQ-VAS containing a scale from 0 to 100, on which the patient records his/her actual health status (0 (the worst you can imagine) and 100 (best health you can imagine). BAROS questionnaire includes three main categories: percentage of excess weight loss, changes in medical conditions, and quality of life. The quality of life questionnaire comprises the following dimensions (self-esteem, physical activity, social contacts, work conditions, sexual activity and eating behaviour) scored by the patient on a 10 point Likert scale

The BARGASTRO study is designed as a prospective, monocentric, randomized, two-arm parallel, active-controlled, superiority trial to compare a new unidirectional mid-term absorbable barbed (UBS) versus an absorbable traditional suture material (CS). A total of 150 patients are enrolled in one German clinic and randomly allocated in a 1:1 ratio to both suture groups. The surgical technique to create the gastro-jejunal as well as the jejuno-jejunal anastomosis will be standardized in both suture groups, and the participating surgeons are trained on the used suture technique. Patients will be kept unaware of the allocation (single blinded) until the completion of the study. After surgery, each subject will be examined on the day of discharge, 30 days, and 12 months postoperatively onsite at the hospital by surgeons performing the surgical procedure. This scheduled time has been selected because it represents the clinical routine setting of the participating hospital. Registration of the study has been performed in the platform for international clinical trials of the World Health Organization under http://www.clinicaltrials.gov NCT05433688 on 12 Oct. 2022. The local medical ethics committee responsible for the participating clinic approved the study on 15 March 2022 under project number D530/21. Our trial is reported in accordance with the SPIRIT guideline, which is a standard to publish study protocols [37]. The SPIRIT checklist is provided as an appendix, and a study flow chart is also included (Fig. 1).

## Methods: participants, interventions, and outcomes

### Study setting {9}

Patients will be recruited in a consecutive manner in one German Academic University Hospital (UKSH, Department of General, Visceral, Thoracic, Transplant and Paediatric Surgery, Kiel, Germany), which is familiar and well trained in the application of barbed and conventional threads as well as in robotic-assisted Roux-en-Y gastric bypass surgery. Approximately, 40,000 patients are treated in this hospital and 2300 within the Department of General, Visceral, Thoracic, Transplantation and Paediatric Surgery, and 80–100 robotic Roux-en-Y gastric bypasses are performed annually, revisional bariatric bypass procedures excluded (10–15 per year). The clinic has been selected to exclude a learning curve.

### Eligibility criteria {10}

#### Inclusion criteria

Patients being at least 18 years of age with a BMI ≥ 40 kg/m^2^ or with a BMI ≥ 35 kg/m^2^ combined with one or more of the following comorbidities: refractory arterial hypertension, type 2 diabetes mellitus, and/or proven sleep apneoa scheduled for an elective, primary robotic-assisted gastric bypass surgery with the need to approximate the gastro-jejunal anastomosis (GJA) and the jejuno-jejunal anastomosis (JJA) were eligible for participation.

#### Exclusion criteria

- Emergency surgery
- Previous gastric surgery
- History of chronic steroid use
- Pregnancy or breastfeeding.
- Patients with hypersensitivity or allergy to the suture material
- Noncompliance
- Participation in another RCT study

### Who will take informed consent? {26a}

Experienced and trained physicians of the Department of General, Visceral, Thoracic, Transplant and Paediatric Surgery, UKSH, Kiel, Germany, will screen the patients for eligibility. Patients who meet the inclusion criteria will be asked for their willingness to participate in the BARGASTRO study and will be informed by the physicians with verbal and written information about the purpose of the study, the operation modalities, and their benefits and risks. The written informed consent has to be obtained from each agreed participant in accordance with the origins of the Declaration of Helsinki and before any study procedure occurs. The participants are recruited several weeks before RYBG surgery.

### Additional consent provisions for collection and use of participant data and biological specimens {26b}

Due to the Data Protection Law (GDPR), which has been effective in Europe since 25 May 2018, a written informed consent has to be provided by each patient for the collection and usage of individual patient data.

## Interventions

### Explanation for the choice of comparators {6b}

An effective construction of the intestinal anastomoses is of major importance. The intestinal anastomosis can be performed using interrupted or continuous sutures or staples to prevent complications. Using suture materials for the loss of tension due to suture loosening is an important issue. To maintain the tension, a constant traction by the assistance and repeated tightening of the suture is necessary. The anchoring properties of a barbed suture material obviate the need for a conventional surgical knot and provide tissue approximation and traction without requiring an assistant, thus improving surgical efficiency. In addition, barbed sutures prevent suture slipping, enable tieless and speedy, and secure anastomosis approximation. This is especially beneficial in minimally invasive procedures, where constraints of space and manoeuvrability may present special technical challenges, especially in robotic surgical interventions, where no assistance controls and pulls the suture material regularly. Therefore, in the present study, the performance of a new unidirectional barbed thread is compared to a conventional one in robotic-assisted RYGB. Both selected suture materials are approved and carry the CE-mark. In addition, both devices are applied in their intended use.

#### Unidirectional barbed suture (UBS) description

**Fig. 2:**
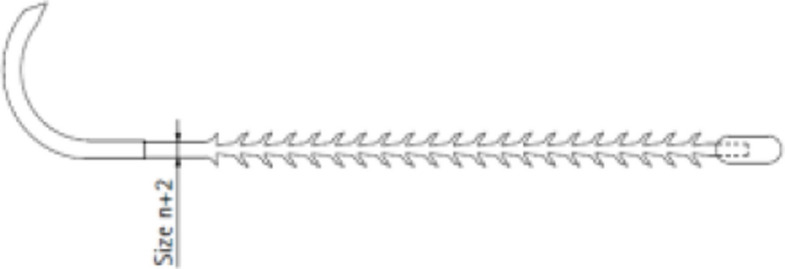
Schematic drawing of the unidirectional configuration of the barbed thread

The barbed suture material investigated in the current study is named Symmcora^®^ mid-term and is manufactured by B. Braun Surgical SA, 08191 Rubi, Barcelona, Spain. It is a sterile synthetic, mid-term absorbable monofilament symmetric anchoring device made from a copolymer of 72% glycolide, 14% *ε*-caprolactone, and 14% trimethylene carbonate, comprising an elongate main body (core) with anchoring elements that provide a knotless wound closure capacity to the device, intended for secure fixation in tissue without using knots. In the current study, the barbed suture material is used in its unidirectional configuration. Here, a single group of anchors is placed along the elongated body, being the wound closure device provided with a locking system at the distal end, opposite to the longitudinal direction the anchors point out (Fig. 2). The diameter refers to the unbarbed section length near the needle attachment area. The diameter in the needle-attachment zone of Size_n_ is equivalent to the diameter of Size_n+2_ having the same USP designation.

Biocompatibility tests have shown that the unidirectional barbed suture is non-cytotoxic, non-mutagenic, non-genotoxic, non-toxic, non-pyrogenic, non-irritating, non-sensitizing, and biocompatible. The suture tensile strength is 87% after 7 days and 51–57% after 14 days. Mass absorption of the device is essentially complete after approximately 90–120 days post-implantation.

#### Conventional suture (CS) description

As a comparative suture material, Novosyn^®^ manufactured by B. Braun Surgical SA, 08191 Rubi, Barcelona, Spain, was chosen. It is a sterile synthetic absorbable braided surgical suture composed of a copolymer made from 90% glycolate and 10% L-lactate. The suture material is metabolized to glycolic and lactic acid by hydrolysis. About 75% of the original tensile strength remains after 14 days of implantation, about 40–50% after 21 days, and about 25% after 28 days. The suture is completely absorbed by hydrolysis within 56–70 days.

### Intervention description {11a}

All RYBG surgeries will be carried out as usual and according to the local standard using the da Vinci Xi Surgical System (Intuitive, Sunnyvale, CA, USA). Gastrojejunostomy (GJ) and jejuno-jejunostomy (JJ) in the Novosyn^®^ Group are performed in the same way as the GJ and JJ of the Symmcora^®^ Group. Randomization occurs after the creation of the side-to-side linear stapler anastomosis. Then, the enterotomy is closed by a continuous, running seromuscular suture. GJ is usually created with two threads starting from the corners. The JJ is usually with one thread. There is no difference regarding the procedure if Novosyn^®^ or Symmcora^®^ is used.

#### Experimental group = unidirectional barbed suture group (UBS)

In the unidirectional barbed suture group (UBS group), two barbed threads of USP 2/0, 15 cm, and violet with a HR 26-mm needle will be applied. The first stitch of the first thread is performed in the left lateral corner of the enterotomy, with the inclusion of the staple line. After completion of one-half of the anastomosis, a second thread is started from the right corner. The last 2–3 stitches with each thread are guided in the opposite direction to anchor the suture in the tissue and to dispense with the need for a knot. The threads are cut at an appropriate length to avoid local adhesions.

Proximal to the anastomosis, the small intestine is separated with a linear stapler. A Roux limb of 150 cm is measured, and a side-to-side linear stapled jejuno-jejunostomy is created again using a linear stapler. The enterotomy is closed by a single running suture using the unidirectional barbed thread of USP 3/0, 15 cm, and violet with a HR 26-mm needle. After completion of the anastomosis, a methylene blue test of the gastrojejunostomy, the check for blood dryness, the repositioning of the omentum majus, and the installation of a drainage are regularly carried out.

#### Control group = conventional suture group (CS group)

To complete the gastrojejunostomy, conventional threads of USP 2/0, 45 cm, violet, and with a HR 26-mm needle are used (two threads), and to complete the jejuno-jejunostomy, one conventional thread of USP 3/0, 45 cm, violet, and with a HR 26-mm needle will be applied. For the gastrojejunostomy and jejuno-jejunostomy, the suture starts and ends with a knot in comparison to the UBS group. Two threads are applied and knotted at the corner of the gastrojejunostomy. Two threads are sutured in the opposite direction and will be knotted together at the middle of the complete suture. The jejuno-jejunostomy is closed with a single running suture.

### Criteria for discontinuing or modifying allocated interventions {11b}

Exit criteria will be as follows: (1) RYGB is not possible to be performed due to severe adhesions, (2) intraoperative decision to perform a gastric sleeve instead of a RYGB, and (3) intraoperative decision to modify the procedure due to visceral obesity, hepatomegaly, or adhesions.

### Strategies to improve adherence to interventions {11c}

Before the start of the trial, an initiation visit was performed in the clinic by the study management to inform and instruct the involved personnel on the study-specific documents and procedures. The surgeons and nurses will document the collected data directly in a paper-based case report form. The documentation is checked on a regular basis by a research monitor to verify adherence to the study protocol and to perform source data verification. Already during the inpatient clinic stay, each patient receives the appointment for the follow-up examination in writing by the study personnel. To standardize both suture groups, suture material from the same batch is packed in the opaque randomization envelopes. After surgery, a randomization fax is sent by the clinic to the sponsor to report the successful inclusion of a patient in combination with the randomization result.

### Relevant concomitant care permitted or prohibited during the trial {11d}

No suture material or suture technique other than described in the protocol can be used for GJA and JJA construction. Any protocol violation has to be clearly described and reported. Postoperative care is performed in accordance with the clinical standard, which includes the following parameters, but these will not be reported and collected in the CRF: same-day surgery, single-shot antibiotic prophylaxis, perioperative pneumatic compression, early mobilization, ulcus prophylaxis (Pantozol 40 mg), thrombosis prophylaxis (Innohep 50 IE/kg), early oral food intake, nutritional advice, intramuscular vitamin B12 substitution, and length of hospital stay ranging from 1 to 4 days.

### Provisions for posttrial care {30}

N/a. Reason: patients are treated in accordance with the clinical routine; all complications occurring during the study will be treated in accordance with the hospital’s treatment protocol.

### Outcomes {12}

Collection of the study outcomes will be performed preoperatively (baseline clinical data, demographics, eligibility), intraoperatively (randomization, closure time, suture handling, surgical details), at day of discharge, 30 days, and 12 months postoperatively. Figure 1 lists the primary and secondary outcomes including the measurement time.

#### Primary outcome

Time (in minutes) to perform the gastro-jejunal and jejuno-jejunal anastomosis using a stopwatch is the primary endpoint. Time starts when the needle passes the tissue the first time and ends when the needle is cut from the thread. The time to perform both the GJA and the JJA (rounded to the nearest full minute) will be the total anastomosis time.

#### Secondary outcomes

The rate of anastomosis leaks, anastomosis stenosis, gastric fistula, obstruction, anastomosis bleeding and re-anastomosis, and the frequency of other complications according to Clavien–Dindo will be collected as safety variables until discharge, 30 days, and 12 months postoperatively [38].

In addition, the operation duration, costs, length of hospital stay, patient satisfaction, pain, and quality of life will be compared in both suture groups and over time.

Length of hospital stay is defined as the period from day of surgery until day of discharge. Cost assessment includes the costs of suture material, costs per operation minute, number of used threads, and costs per hospital day. Pain evaluation will be performed by the patient using the visual analogue scale (VAS), which is a numeric scale ranging from 0 (no pain) to 100 (maximal pain). In addition, patients will be asked by the surgeon for their postoperative satisfaction using a numeric scale from 0 (not satisfied) to 100 (maximal satisfaction). Quality of life will be analysed using the EQ-5D-5L questionnaire, which is a standardized measure of health status developed by the EuroQol Group to provide a simple, generic measure of health for clinical and economic appraisal [39]. The questionnaire consists of a descriptive system comprising five dimensions (mobility, self-care, usual activities, pain or discomfort, anxiety, or depression) and the EQ visual analogue scale (EQ-VAS). Each dimension has five levels: 1 = no problem, 2 = slight problems, 3 = moderate problems, 4 = severe problems, and 5 = extreme problems. The result of the assessment of all five dimensions leads to the so-called EQ index, which ranges from 1 minimum to 5 maximum. Using the EQ-VAS, each patient records his/her healthy status ranging from 0 (the worst health you can imagine) to 100 (best health you can imagine). The EQ5D5L is used in the German language, and a licence has been obtained by the sponsor from EuroQol–Group. 

Furthermore, the Bariatric Analysis and Reporting Outcome System (BAROS), which is a simple, objective, unbiased, and standardized method to analyse and report outcomes of bariatric surgery, is used in the current study [40]. The BAROS questionnaire consists of a scoring table that comprises three columns with the main areas of interest: weight loss (% of excess), improvement of medical conditions, and quality of life. A maximum of 3 points can be given in each domain to judge the changes after surgery. Weight loss is scored as follows: If the final weight is higher than before surgery, a full point is deducted; no point is assigned if the excess weight loss is between 0% and 24%, 1 point is given for a weight loss between 25 and 49%, 2 points for a weight loss between 50 and 74%, and 3 points for a weight loss between 75 and 100%. Regarding the category “improvement of medical conditions”, the changes after surgery can be scored using the following categories: unchanged = no point, improved = 1 point, one major condition resolved others improved = 2 points, all major conditions resolved, and others improved = 3 points; if medical conditions are aggravated after surgery, 1 point is deducted. The QoL domain of BAROS includes a specially designed patient questionnaire (the Moorehead-Ardelt Quality of Life Questionnaire II) that uses simple drawings to score on a 10-point Likert scale each of the six QoL questions: self-esteem, physical activity, social contacts, work conditions, sexual activity, and eating behaviour. The questionnaire is equally weighted, and points are added or subtracted in accordance with the patient’s response. Ratings on the right side of the questionnaire reflect positive changes after surgery, leading to the assignment of partial points, whereas assessments on the left side of the questionnaire reflect negative changes, which reduce the total subscore of QoL. After completion of the assessment of all three domains, the subtotals are added; but before the final score is calculated, points are deducted for complications or reoperations without any qualification of these. In the case of a major complication or a reoperation, 1 point is subtracted, whereas a minor complication leads to a subtraction of 0.2 points. Based on a scoring table that adds or subtracts points while evaluating the three main categories: percentage of excess weight loss, changes in medical conditions, and quality of life (QoL), the final score defines five outcome groups: failure (1 point or less), fair (> 1 to 3 points), good (> 3 to 5 points), very good (> 5 to 7 points), and excellent (> 7 to 9 points), reflecting the failure or success of the treatment. BAROS is used in the German version, and a licence for usage was issued to the sponsor by M. K. Moorehead.

Handling of the suture material will be judged by the participating surgeons after each surgery using a questionnaire including different categories with five evaluation levels (excellent, very good, good, satisfied, poor), and the outcome for both suture types (barbed vs. conventional) will be compared. The following categories will be assessed in the case of the conventional suture material (pliability, tissue drag, knot security, knot run down, knot pull tensile strength, anastomosis approximation, overall impression). The assessment of the barbed suture includes the following dimensions: pliability, pass through the tissue, traumaticity of the barbs, anchoring capacity of the barbs, perception of the locking system, anastomosis approximation, and overall impression.

### Participant timeline {13}

An overview of the time schedule and study measurements per visit is shown in Fig. 1.

### Sample size {14}

The sample size calculation is based on the primary endpoint “time to perform the GJA and JJA” to show superiority of a unidirectional barbed suture compared to a conventional suture material. Several studies have been published analysing both types of suture materials regarding this outcome parameter [6, 7]. The weighted group means and the weighted pooled standard deviations were calculated based on the results of the subset of four studies [24–27] providing suturing times to create the GJA. Considering 14.20 min to approximate the GJA in the barbed suture group compared to 16.10 min in the conventional group, a sample size of 71 patients in each suture group will have 80% power to detect a mean difference of −1.90 min assuming that the common standard deviation is 4.00 using a two-sample *t*-test with a 0.05 two-sided significance level. Including a drop-out rate of 5%, the sample size becomes up to 75 patients in each suture group, a total of 150 patients. The times to create the JJA are only reported in two studies [25, 27] with great differences. Therefore, to calculate the sample size for the current study, the time for the construction of the JJA was not taken into account. In addition, it was previously mentioned that the times for JJA formation did not differ between traditional and barbed sutures [6]. For sample size calculation, nQuery Advisor^®^7.0 was used.

### Recruitment {15}

In total, 150 patients will be recruited in one German academic university hospital. Each patient who is scheduled for a Roux-en-Y bypass will be informed about the study and will be asked for his/her willingness to participate in the study to reach the target sample size. The total study duration is planned for 3 years (2 years for recruitment plus 1 year for the follow-up).

## Assignment of interventions: allocation

### Sequence generation {16a}

A statistician of the sponsor has prepared a computer-generated randomization list equally distributing both suture groups in a 1:1 ratio using different random block lengths unavailable to the clinic. The block sizes will not be disclosed, to ensure concealment. The randomization list is sealed and locked up at the sponsor site. Thus, the medical staff has no influence on the randomization. A stratification based on risk factors was not performed.

### Concealment mechanism {16b}

Patients will be randomized intraoperatively using opaque, sealed envelopes, containing the information of the random allocation of the device, a randomization fax, and the respective suture material (UBS or CS) in appropriate numbers and in different USP sizes to create the GJA as well as the JJA. The envelopes are prepared by the sponsor and provided to the clinic in sufficient numbers. The randomization envelopes are stored in the principal investigator’s office and individually carried to the operation theater for each surgery by the responsible surgeon. Assignment to the patients will be done in a chronological manner by the surgeon, according to a consecutive random number. Briefly after the construction of the GJA using the 45-mm linear stapler before suturing the enterotomy, the nurse will open the envelope and mention the randomization result to the operation team. After each surgery, the clinic has to send the included randomization fax to the sponsor to confirm the inclusion of a patient as well as the randomization result.

### Implementation {16c}

Before the start of the trial, an initiation visit was performed by the project management of the sponsor to inform and to instruct the involved medical staff on the specific study documents and procedures.

## Assignment of interventions: blinding

### Who will be blinded {17a}

Patients will be unaware of the type of suture material they receive to ensure a valid assessment of the postoperative outcome parameter, which they judge. It is impossible to blind the surgeon because, first, the suture type can be differentiated by configuration and feel, and second, the surgeon has to be aware of the suture type to perform the anastomosis. The statistician has no access to the eCRF or database. After data base closure, generation of the data export (performed by the data manager), and determination of the analysis population, the statistician responsible for the analysis will be unblinded.

### Procedure for unblinding if needed {17b}

An unblinding of the patients is not planned during the study, but if the patient has to be unblinded, e.g. because of serious adverse events, the patient will stay in the study and be analysed as planned. If the number of unblinded patients would be too high, maybe a subgroup analysis has to be performed, comparing blinded and unblinded patients.

## Data collection and management

### Plans for assessment and collection of outcomes {18a}

All participant data required based on the study protocol will be collected by the principal investigator or trained representatives and entered in a paper-based CRF provided by the sponsor.

Intraoperative documentation includes the following—the surgeon’s initials, surgeon’s position, randomization result (UBS or CS), and the use of antibiotic prophylaxis—and is performed for all study subjects.

In addition, the following parameters are mandatory and recorded and measured in all randomized patients during surgery:

- Total operation duration
- Estimated blood loss
- Intraoperative blood transfusion
- Suturing time to create the gastro-jejunal anastomosis
- Suturing time to create the jejuno-jejunal anastomosis
- Number of used threads
- Outcome of the leak test
- Handling assessment of the suture material
- Device malfunctions
- Adverse events

The EQ-5D-5L and BAROS self-assessment quality-of-life questionnaires will be filled in by the patients themselves. The completed CRFs and questionnaires will be securely stored in the principal investigator’s office, which is protected against unauthorized access.

### Plans to promote participant retention and complete follow-up {18b}

The schedule of the study visits corresponds to the standardized aftercare scheme of the hospital. To promote the adhesion of the patients, they are reminded by the involved study nurse of the follow-up appointments by telephone call. The collected data set of patients discontinuing the study prematurely will be analysed until their discontinuation. The reason for discontinuation (e.g. withdrawal from the trial, lost to follow-up) will be recorded.

### Data management {19}

The collected data recorded by the clinic on paper-based CRFs and by the patients on paper-based QoL questionnaires (EQ-5D-5L and BAROS) will be entered by the sponsor’s data management department in a timely manner into an electronic capture system (secutrial) owned by the sponsor. The data entry will be done after the data set has been checked by an authorized research monitor. After each monitoring visit, the responsible research monitor will forward the original paper-based CRF and the original paper-based EQ-5D-5L and BAROS questionnaires to the sponsor’s data management for data entry. The data entry will be performed by the responsible data managers (two different persons), not by the involved statistician. To ensure data quality and reliability, a double entry will be performed by the data management. Thereafter, the entered data will be checked for completeness, correctness, plausibility, and consistency by validating programmes, which thereby produce queries. All inconsistencies will be forwarded to the responsible investigator for clarification and explanation. The original CRFs will be stored at the sponsor’s site, and a copy will remain at the clinic’s site. The data will be archived for 10 years.

### Confidentiality {27}

Patient data will be handled confidentially and subjected to the data protection law. The principal investigator ensures that CRF or questionnaires transmitted to the sponsor do not contain personal patient data. Each patient will receive an individual patient study identification number (pseudonymization), and only the investigator will be able to identify a patient by the unique patient study identification number. A separate patient identification list will be set up and stored confidentially at the clinic site.

### Plans for collection, laboratory evaluation, and storage of biological specimens for genetic or molecular analysis in this trial/future use {33}

Not applicable, because no biological specimens will be collected in this study.

## Statistical methods

### Statistical methods for primary and secondary outcomes {20a}

The intention-to-treat principle will be applied for data analysis. A CONSORT diagram will be utilized to graphically display the eligibility, allocation, and follow-up course of the participants (**41**). Demographics, anamnesis, and baseline characteristics will be presented in a tabular form by treatment group. Continuous measures will be summarized by the number of non-missing observations, minimum, maximum, median, and mean with standard deviation, and categorical measures by absolute and relative frequencies.

The primary outcome measure is the suturing time to perform the GJA and JJA. The independent samples *t*-test with a two-sided significance level of 0.05 will be used for the comparison of treatment groups (UBS vs. CS). 95% CI will be provided to allow a quantitative evaluation of the treatment effect with respect to the minimum clinically relevant difference of 1.9 min. The secondary variables will be analysed descriptively.

The test regarding the primary variable is considered confirmatory; all other tests are explanatory. For the analysis of binary data, a chi-square test will be used; for nonparametric data, a *U*-test according to Wilcoxon–Mann–Whitney or to Kruskal–Wallis will be used; and a *t*-test or one-way ANOVA will be used for metric data, if a normal distribution is assumed. All statistical tests will be performed two-tailed with a pre-specified significance level of *α* = 5%. Two-sided 95% confidence intervals will be given, where appropriate.

The secondary outcomes include the following:

- Pain (visual analogue scale, values from 0 to 100, 100—max. pain)
- Satisfaction (numeric scale values from 0 to 100, 100—best)
- EQ-5D-5L questionnaire: EQ index range from 1 best to 5 worse and EQ-VAS scale value (0–100—best).
- BAROS questionnaire, three sub-scores with a maximum of 3 points each (weight loss (% of excess), improvement of medical conditions and Quality of Life), and the total score with a maximum of 9 points (best).
- Length of hospital stay in days
- Handling assessment of the suture material (different dimensions using a Likert scale with levels from 1 excellent to 5 worse).

The outcomes will be analysed descriptively and compared between suture groups and between visits, where appropriate.

### Interim analyses {21b}

The main analysis will be done after the completion of the 12-month follow-up. No interim analysis will be carried out before the study is completed.

### Methods for additional analyses (e.g. subgroup analyses) {20b}

To identify relevant influencing factors (confounders), multivariate regression models will be considered. Depending on the outcome variable, linear or logistic models will be implemented, and the patient’s age, sex, BMI, and respective baseline values will be used as covariates to adjust for.

### Methods in analysis to handle protocol non-adherence and any statistical methods to handle missing data {20c}

All patients without a violation of the inclusion and exclusion criteria and who received the intended study treatment will be included in the primary analysis. Missing data/values will be analysed as such and will not be replaced by estimates. When reporting statistical distribution parameters, the number of non-missing values will be provided for transparency. Deviation from the study protocol will be assessed as protocol violations. If at least one inclusion or exclusion criterion is not fulfilled, the patient will be dropped out from the study.

### Plans to give access to the full protocol, participant-level data, and statistical code {31c}

Not applicable. Due to regulations and the data protection law, only the sponsor of the study and the participating clinic will have access to these details.

## Oversight and monitoring

### Composition of the coordinating centre and trial steering committee {5d}

To ensure adherence to the study protocol, accuracy of the reported data, and protection of patient rights and to assess performance of the participating site, regular monitoring visits will be performed by qualified authorized representatives of the sponsor (monitor). To verify source data, the monitor will have access to the data and source documents. Up to 3–4 monitoring visits are planned annually in the participating clinic, and a monitoring plan has been set up in advance. After each monitoring visit, the monitor transfers the completed CRF to the data management of the sponsor for data entry, and in addition, a monitoring report summarizing all actions and findings will be generated by the monitor.

The Department of General Surgery of the University Hospital in Kiel is responsible for patient recruitment, collection of the written informed consent, conduct of surgical interventions, treatment allocation of the participants, clinical data collection, and adverse events reporting. The project management, monitoring, statistical analysis, data management, and registration of the study are under the responsibility of the sponsor, which also provides the funding of the trial. A final study report as well as the final results will be generated and published by the participating clinic in cooperation with the sponsor. The publication of the results according to CONSORT statement is expected to take place in early 2026 [41].

### Composition of the data monitoring committee and its role and reporting structure {21a}

A formal data monitoring committee is not required for the setup, since the study analyses two approved suture materials applied within their intended use.

### Adverse event reporting and harms {22}

All adverse events (AE), serious adverse events (SAE), and device deficiencies (DD) have to be reported by the clinic using the AE/SAE/DD form, which is integrated into the CRF, to the sponsor within 24 h–48 h after occurrence. In addition, for each event, the seriousness, intensity, expectedness (expected/unexpected), and causal relationship with the device or the surgical procedure have to be recorded, including the measures taken as well as the outcome of each event. Unexpected serious adverse events have to be notified by the sponsor to the responsible EC after acknowledgement. For documentation and reporting to the EC, the MDCG Guidance 2020-10/1 Rev1 and the SAE report form 2020-10/2 Rev1 provided for the safety reporting in clinical investigations will be utilized (0537d335-7eed-4087-b65d-3c2bd8c72c1a_in (*europa.eu*). Events reported with a suspected or proven causal relationship with the device will be additionally reported to the Quality Management Department of the sponsor according to vigilance processes. All events occurring until the patient’s 12-month postoperative examination will be documented, and ongoing events will be followed up until 28 days following the patient’s last visit.

The following events will be reported as adverse and/or serious adverse events:

- Anastomosis leak
- Anastomosis bleeding
- Anastomosis stenosis
- Gastric fistula
- Obstruction
- Reoperation due to complication of the anastomosis
- Death
- Any complication with the need of a nonoperative or operative measure to prevent a life-threatening injury or impairment to the body structure or body function
- Any complication leading to hospitalization or hospitalization prolongation

The following events will be reported as adverse and serious device events:

- Suture rupture
- Needle bending
- Disconnection between the needle and the thread
- Disconnection between the fixation element (anchor) and the residual barbed suture
- Any device-related complication

Device deficiency is defined as an inadequacy of the medical device with respect to its identity, quality, durability, reliability, usability, safety, or performance. This definition includes device deficiencies related to the experimental group (UBS) or the control group (CS).

### Frequency and plans for auditing trial conduct {23}

Audits are not planned by the sponsor because all surgical interventions and follow-up examinations are performed in clinical routine. In addition, the clinic is a well-trained and experienced high-volume centre with regard to the operations carried out in the current study. In the event that an audit would be performed by an independent external party, the clinic has given advance consent to provide the responsible persons with access to the data and all documents, which are needed for the inspection.

### Plans for communicating important protocol amendments to relevant parties (e.g. trial participants and ethical committees) {25}

If essential modifications are necessary to the original study documents, an amendment will be generated by the sponsor in cooperation and agreement with the principal investigator of the clinic and submitted to the responsible ethics committee for approval in line with pertinent regulatory requirements. The investigator, as well as the clinic, will not implement any deviations or changes without mutual agreement, prior review, and approval of the respective ethics committee. Changes will be also reported to trial registries, to journals, and to participants if they are affected by these modifications.

### Dissemination plans {31a}

The final results of the study will be published in an international peer-reviewed open-access journal to reach a high readership of healthcare professionals. The respective reference will be listed in trial registration under www.clinicaltrials.gov. In addition, it is planned to present the outcome of the study in national and international congresses.

## Discussion

Minimally invasive surgery has become the standard procedure for different surgical interventions; this is also the case for general surgery because of its advantages regarding reduced pain and faster recovery compared to open surgeries [1, 9]. In general surgery, the most challenging and time-consuming step is the formation of the intracorporeal sutured anastomosis [2, 3, 6, 7]. Barbed suture materials offer some advantages in comparison to standard sutures for minimally invasive surgery because these sutures do not require knot-tying, and they prevent the potential of suture slipping during continuous suturing [6, 7, 10]. A total of four meta-analyses have been published, analysing the benefits of barbed sutures compared to conventional suture materials for the construction of laparoscopic gastrointestinal anastomosis [6–9]. The authors demonstrated a reduction of operation time and anastomosis time for barbed sutures, whereas the rates for anastomosis leak, anastomosis stricture, and anastomosis bleeding were comparable in both suture groups [6–9]. Barbed suture materials are safe with an equivalent complication profile compared to standard suture materials [6, 9]. However, there is less evidence on the role of barbed sutures in the formation of anastomoses during robot-assisted procedures. While the use of sutures in robotic radical prostatectomies has been established and studied [42], to our knowledge, there are no RCTs or meta-analyses on the use of barbed sutures in robotic bariatric surgery. Nevertheless, their use in robotic bariatric surgery is already widespread.

The widespread usage of barbed suture in minimally invasive surgery is mainly limited due to its high material costs. Only a few studies assessed the cost difference between standard and barbed sutures’ materials. A cost-saving was reported because the additional material costs could be compensated by the significant reduction of operation time seen with barbed sutures [8, 43, 44]. In bariatric minimally invasive surgery, the operation time appears to be particularly related to the GJA, whereas no significant difference was observed for the JJA [6, 7]. Different anastomosis techniques are applied [45, 46]. In addition to circular anastomoses, which do not require additional sutures, anastomoses have been created using linear staplers or a hand-sewn technique for a long time. While the linear stapled anastomosis is preferred laparoscopically [47], robotically hand-sewn anastomoses are frequently used. These procedures appear to be more time consuming but may have advantages in terms of marginal ulcers [48].

Obesity is a global disease with increasing numbers worldwide [49, 50]. Bariatric interventions, such as Roux-en-Y bypass as well as sleeve gastrectomy, are effective surgical procedures to reduce weight loss and comorbidities [51–53]. Barbed sutures have been confirmed as safer, easier to handle, and more reproducible without any observed anastomosis-related complications since their first use for gastrointestinal enterotomy [30].

The use of the robot in bariatric surgery continues to be controversial. Minimally invasive bariatric surgery is already highly standardized and inherently associated with low complication rates [54], making it difficult to further improve the excellent results with the help of robotic surgery. All studies report that robotic bariatric surgery is safe and efficient. The results are comparable to laparoscopic surgery but are associated with higher costs and seemingly longer operative times when using the robot [42, 55, 56]. Recent studies show slight advantages in terms of postoperative complications in favour of robotic surgery [48]. In the participating clinic, gastric bypass surgery and bariatric revisional procedures are performed robotically as standard. Since using the surgical robot, operation times and inpatient hospitalization times have been significantly reduced, with lower complication rates compared to the operations previously performed laparoscopically [57].

In the present study, a robotic-assisted Roux-en-Y gastric bypass was chosen to treat obesity as a standard surgical procedure in the participating clinic. GJA and JJA are performed using a 45-mm linear stapler. The linear GJA and JJA are the in-house standard due to the high standardization and shorter operative time compared to a hand-sewn anastomosis. The outcome regarding safety and performance using different parameters will be compared in patients randomly receiving either a barbed suture material or a conventional one for gastro-jejunal anastomosis and jejuno-jejunal anastomosis formation.

The current BARGASTRO study will be as follows:

- First: Analyse a new unidirectional mid-term absorbable barbed suture material, which differs in its configuration from other existing ones (e.g. anchor instead of a loop for fixation, parallel-oriented barbs instead of spiral or linear offset barbs, shorter barbs, and higher smoothness).
- Second: Be the first and largest RCT so far performed in robotic-assisted Roux-en-Y gastric bypass surgery comparing barbed versus conventional sutures for the formation of GJA and JJA
- Third: Report the anastomosis time for GJA and JJA separately, because data on JJA are limited.
- Fourth: Provide clinical evidence on level 1b, because of its high-quality, randomized-controlled, large sample size, patient-blinded, and long-term follow-up design.

## Trial status

The study was reviewed and approved by the responsible ethics committee of the UKSH, Kiel, Germany, on 15 March 2022. Initiation of the clinic was done on 8 June 2022. Registration of the trial under www.clinnicaltrials.gov was made on 27 June 2022, NCT05433688; study details are Study on the Performance of Symmcora^®^ mid-term suture versus Novosyn^®^ suture in patients undergoing robotic-assisted gastric bypass surgery, ClinicalTrials.gov. Randomization and surgical intervention of the first patient occurred on 12 October 2022. During the preparation of the manuscript, two-thirds of patients had been randomized, and 33 patients had completed their 12-month postoperative examination. End of recruitment is planned for March 2025, and completion of the study, including the 12-month follow-up visit of the last randomized patient, is expected by the first quarter of 2026.

## Data Availability

Due to regulatory issues, data availability of the current study is limited and only provided on reasonable request from the sponsor. Only the sponsor and the clinic will have full access to the final data set. The results from this study will be published in an open-access peer-reviewed journal to provide a high distribution, transparency, and readership.

## Abbreviations

AE: Adverse event
ASA: American Society of Anesthesiologists
BAROS: Bariatric Analysis and Reporting Outcome System
BARGASTRO: BARbed suture to close GASTROintestinal anastomosis
BMI: Body mass index
CRF: Case report form
CS: Conventional suture
DD: Device deficiency
EC: Ethics committee
EQ-5D-5L: Euro Quality of Life questionnaire
GJA: Gastro-jejunal anastomosis
ID: Identification number
ITT: Intention to treat
JJA: Jejuno-jejunal anastomosis
Max: Maximum
MDCG: Medical Device Coordination Group
Min: Minimum
QoL: Quality of life
RYGB: Roux-en-Y gastric bypass
SAE: Serious adverse event
UBS: Unidirectional barbed suture
VAS: Visual analogue scale
